# Marked Sexual Dimorphism in the Role of the Ryanodine Receptor in a Model of Pain Chronification in the Rat

**DOI:** 10.1038/srep31221

**Published:** 2016-08-08

**Authors:** Luiz F. Ferrari, Eugen V. Khomula, Dionéia Araldi, Jon D. Levine

**Affiliations:** 1 Departments of Medicine and Oral Surgery, and Division of Neuroscience, University of California at San Francisco, 521 Parnassus Avenue, San Francisco, CA 94143, USA

## Abstract

Hyperalgesic priming, an estrogen dependent model of the transition to chronic pain, produced by agonists at receptors that activate protein kinase C epsilon (PKCε), occurs in male but not in female rats. However, activation of second messengers downstream of PKCε, such as the ryanodine receptor, induces priming in both sexes. Since estrogen regulates intracellular calcium, we investigated the interaction between estrogen and ryanodine in the susceptibility to develop priming in females. The lowest dose of ryanodine able to induce priming in females (1 pg) is 1/100,000^th^ that needed in males (100 ng), an effect dependent on the activation of ryanodine receptors. Treatment of female rats with antisense to estrogen receptor alpha (ERα), but not beta (ERβ), mRNA, prevented the induction of priming by low dose ryanodine, and the ERα agonist, PPT, induced ryanodine receptor-dependent priming. *In vitro* application of ryanodine in low concentration (2 nM) to small DRG neurons cultured from females, significantly potentiated calcium release via ryanodine receptors induced by caffeine. This effect was only observed in IB4+ neurons, cultured in the presence of β-estradiol or PPT. Our results demonstrate a profound regulatory role of ERα in ryanodine receptor-dependent transition to chronic pain.

While many recurrent, persistent and chronic clinical pain syndromes are more common and/or severe in women (e.g., migraine, irritable bowel syndrome, fibromyalgia, rheumatoid arthritis, and chronic postoperative and neuropathic pain^1^,^2^,^3^,^4^,^5^,^6^), the underlying mechanisms remain poorly understood. We developed a preclinical model of the transition to chronic pain, hyperalgesic priming, in which there is an enhanced responsiveness of isolectin B4-positive (IB4+) nociceptors to hyperalgesic agents, such as prostaglandin E_2_ (PGE_2_), adenosine and serotonin^7^,^8^,^9^,^10^. Although in their absence no hyperalgesia is observed, the exposure of primed nociceptors to these agents induces a prolonged decrease in the mechanical threshold when compared to the non-primed state, due to changes in second messenger signaling^7^,^9^,^11^,^12^. Hyperalgesic priming is highly sexually dimorphic^13^, occurring in male, but not female rats, and it can be produced by peripheral administration of cytokines^7^,^14^ and neurotrophic factors^15^, whose cognate receptors have in common that they acutely sensitize nociceptors, to produce mechanical hyperalgesia^14^,^16^,^17^, mediated at least in part by protein kinase C epsilon (PKCε). A selective activator of PKCε, ψεRACK^7^, also produces hyperalgesic priming in male but not female rats^13^.

Ovariectomy converts the hyperalgesic priming phenotype observed in the female rat to the male phenotype, and estrogen administration reconstitutes the phenotype observed in the gonad intact female^13^. Conversely, administration of estrogen to male rats induces a female phenotype^13^. While these data support the suggestion that sexual dimorphism in hyperalgesic priming is estrogen-dependent, the receptor on the nociceptor at which estrogen acts to produce sexual dimorphism remains to be established.

Activation of mechanisms downstream of PKCε in the induction of priming, e.g. by ryanodine – modulator of the ryanodine receptor, which can release calcium from the endoplasmic reticulum at low concentration^18^,^19^ – and calcium/calmodulin-dependent protein kinase II alpha (αCaMKII), induces priming in both sexes^20^. However, whether there is sexual dimorphism in mechanisms distal to PKCε has yet to be explored.

In the present experiments we evaluated for the presence of sexual dimorphism in one of the mechanisms downstream of PKCε for the induction of hyperalgesic priming, namely that induced by ryanodine. And, since estrogen regulates intracellular calcium homeostasis^21^,^22^, as well as priming, we determined if any dependence of sex differences in ryanodine-induced priming was dependent on the action of estrogen at either of its classic receptors, estrogen receptor alpha (ERα) and/or estrogen receptor beta (ERβ), both present in nociceptors^23^,^24^.

## Results

**Figure 1:**
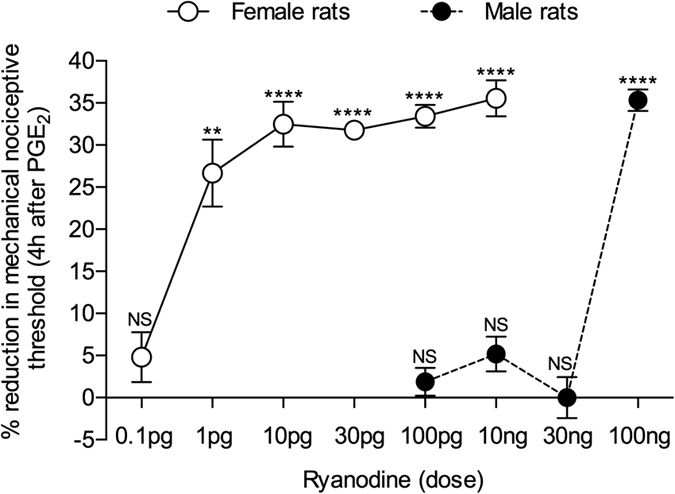
Dose response relationship for ryanodine-induced hyperalgesic priming in male and female rats. Different doses of ryanodine were injected on the dorsum of the hind paw in different groups of female (open circles; 0.1 pg; 1 pg; 10 pg; 30 pg; 100 pg; 10 ng) and male (black circles; 100 pg; 10 ng; 30 ng; 100 ng) rats. No change in the mechanical nociceptive threshold was observed after injections of ryanodine (see Supplementary Fig. S1). PGE_2_ (100 ng) was injected at the same site, 5 days later, and the mechanical hyperalgesia was evaluated by the Randall-Sellitto paw-withdrawal test. Importantly, no difference in the mechanical nociceptive threshold, compared to the baseline mechanical threshold (before injection of ryanodine) was observed (see Supplementary Table S1). The figure shows the mechanical hyperalgesia at the 4^th^ h after the injection of PGE_2_; the presence of hyperalgesia at this time point was used to confirm the induction of priming by the previous treatment with ryanodine. In the groups of female rats that had received doses of ryanodine 1 pg and higher, but not 0.1 pg, and in the group of male rats previously treated with 100 ng of ryanodine, but not with 100 pg, 10 ng or 30 ng, the hyperalgesia induced by PGE_2_ was still present at the 4^th^ h (female rats: 0.1 pg, *t*_5_ = 1.170, *p* = 0.1479 (NS); 1 pg, *t*_5_ = 5.509, *p* = 0.0027 (**); 10 pg, *t*_5_ = 11.59, *p* < 0.0001 (****); 30 pg, *t*_5_ = 20.07, *p* < 0.0001 (****); 100 pg, *t*_5_ = 12.25, *p* < 0.0001 (****); 10 ng, *t*_5_ = 15.41, *p* < 0.0001 (****); male rats: 100 pg, *t*_5_ = 1.151, *p* = 0.3019 (NS); 10 ng, *t*_5_ = 2.534, *p* = 0.0522 (NS); 30 ng, *t*_5_ = 0.0, *p* > 0.9999 (NS); 100 ng, *t*_5_ = 28.76, *p* < 0.0001 (****), when the mechanical nociceptive thresholds before and 4 h after the injection of PGE_2_, for each group, are compared, paired Student’s *t*-test). These results indicate that nociceptors in the female are significantly more sensitive to induction of priming by ryanodine, since a dose much lower was required to induce priming in the female rat. (N = 6 paws per group).

Since estrogen-dependent sexual dimorphism in mechanisms for calcium homeostasis has been demonstrated in diverse cell types^21^,^22^, including in neurons^25^,^26^, we tested the hypothesis that the observed resistance to priming induced by activation of PKCε in female rats^13^, was due to the requirement for more intense stimuli to activate second messenger signaling pathways downstream of PKCε (e.q., a higher concentration of ryanodine could induce priming in the female rat). In marked contrast to our initial hypothesis, we found that ryanodine induces priming in the female rat, expressed as prolongation of the hyperalgesia induced by PGE_2_, observed 4 h after its injection^12^,^20^, at a markedly *lower* dose than needed to induce priming in the male. Thus, a dose 1/100,000^th^ that needed to induce priming in male rats was able to induce priming in females (Fig. 1).

**Figure 2:**
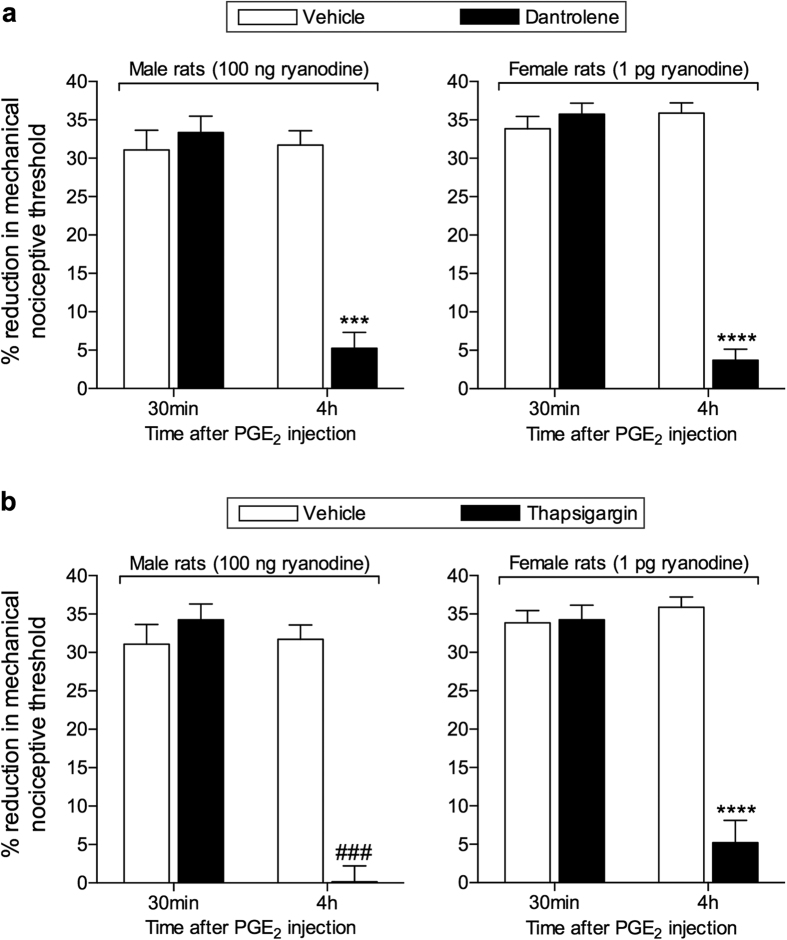
Induction of hyperalgesic priming by ryanodine is dependent on the ryanodine receptor. The ryanodine receptor antagonist dantrolene (1 μg, upper panels, black bars), or the endoplasmic reticulum calcium pump inhibitor thapsigargin (1 μg, lower pannels, black bars), or their respective vehicles (white bars) were injected on the dorsum of the hind paw of male (left panels) and female (right panels) rats. 10 min later, the smallest doses of ryanodine that induced priming (100 ng in male and 1 pg in female) were injected at the same site as the inhibitor or its vehicle. No significant change in the mechanical nociceptive threshold was observed after injection of ryanodine (see Supplementary Fig. S1). Five days later, testing for the presence of priming was performed by intradermal injection of PGE_2_ (100 ng) at the same site as ryanodine and evaluation of the mechanical hyperalgesia, by the Randall-Selitto paw withdrawal test, 30 min and 4 h later. No significant difference (NS) in the mechanical thresholds before the injection of ryanodine and before injection of PGE_2_ was observed (see Supplementary Table S1). Two-way repeated measures ANOVA followed by Bonferroni *post-hoc* test showed that although there was no difference in the hyperalgesia induced by PGE_2_ 30 min after the injection in the vehicle- and inhibitors-treated groups (NS for all groups), at the 4^th^ h its magnitude was significantly smaller in the groups that received the inhibitors before ryanodine injection, 5 days previously (upper panels, males: *F*_1,10_ = 33.10; ***p = 0.0002; females: *F*_1,10_ = 97.21; ****p < 0.0001; upper panels, males: *F*_1,10_ = 27.97; ^###^*p* = 0.0004; females: *F*_1,10_ = 41.83; ****p < 0.0001, when the hyperalgesia in the vehicle- and inhibitors-treated groups is compared at the 4^th^ h), indicating that the induction of priming by ryanodine is dependent on the activation of ryanodine receptor. (N = 6 paws per group).

Ryanodine can block as well as activate the ryanodine receptor^27^, a calcium permeable ion channel^28^,^29^. Since we have previously shown that the hyperalgesic priming induced by ryanodine is dependent on calcium release^20^, probably leading to the activation of CaMKII, in order to confirm that the induction of priming by ryanodine is due to the release of calcium by activation of ryanodine receptors in the endoplasmic reticulum, we next determined if administration of the ryanodine receptor antagonist dantrolene, or thapsigargin, an inhibitor of the calcium pump in the intracellular membrane located in endoplasmic reticulum^30^,^31^, could prevent ryanodine-induced hyperalgesic priming. Pretreatment with either dantrolene or thapsigargin (both 1 μg, intradermally on the dorsum of the hind paw), just prior to injection of ryanodine (100 ng in male, and 1 pg in female rats, at the same site), prevented the induction of priming (Fig. 2). These results support the suggestion that ryanodine induces hyperalgesic priming by its ability to impact the release of calcium from intracellular stores in the endoplasmic reticulum in the peripheral terminal of the nociceptor.

**Figure 3:**
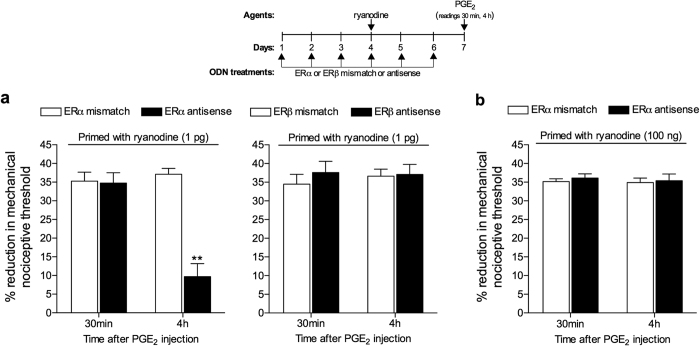
Estrogen receptor alpha (ERα) regulates the induction of hyperalgesic priming by ryanodine in female rats. (**a**) Female rats were treated with ODN antisense (black bars) or mismatch (white bars) for estrogen receptor alpha (ERα, left panel) or beta (ERβ, right panel), for 6 consecutive days. Ryanodine (1 pg) was injected on the dorsum of the hind paw on the 4^th^ day of ODN treatment. On the 7^th^ day, PGE_2_ (100 ng) was injected at the same site as ryanodine, and the mechanical nociceptive threshold was evaluated, 30 min and 4 h later. No difference (NS) was observed in the mechanical nociceptive thresholds before the injection of ryanodine and immediately before injection of PGE_2_ (see Supplementary Table S1). PGE_2_-induced hyperalgesia was still present 4 h after injection in all groups, except in the group treated with ODN antisense for ERα (*F*_1,10_ = 16.22; **p = 0.0024, when the groups treated with ERα ODN are compared; *F*_1,10_ = 0.2610; *p* =s0.6205, NS, when the groups treated with ERβ ODN are compared; two-way repeated measures ANOVA followed by Bonferroni *post-hoc* test); (**b**) The dose of ryanodine that induced priming in male rats (100 ng) was injected on the dorsum of the hind paw of female rats that have been treated intrathecally with ODN antisense or mismatch for ERα. The ODN treatment was performed for 6 consecutive days, and ryanodine was injected on the 4^th^ day. PGE_2_ (100 ng) was injected at the same site as ryanodine on the 7^th^ day, and the mechanical hyperalgesia was evaluated 30 min and 4 h later. No significant difference in the mechanical thresholds before the injection of ryanodine and before injection of PGE_2_ was observed (see Supplementary Table S1). We found that the hyperalgesia induced by PGE_2_ was still present 4 h after injection, with no attenuation, in both groups (*F*_1,10_ = 0.3839; *p* = 0.5494, NS, when both groups are compared). Together, these results support the suggestion that ERα regulates the capacity of ryanodine to induce priming in the female rat. (N = 6 paws, all groups).

To determine which receptor mediated the estrogen-dependent sexual dimorphism in ryanodine-induced priming^12^,^20^ we administered oligodeoxynucleotides (ODN) antisense (AS) or mismatch (MM) to the mRNA for ERα or ERβ, intrathecally to the lumbar spinal cord, in female rats. The ODNs were administered starting 3 days prior to the induction of priming by the intradermal injection of ryanodine, on the dorsum of the hind paw, and continued for 3 days after ryanodine administration. We found that treatment with the AS ODN to ERα, but not to ERβ mRNA, prevented the induction of hyperalgesic priming in the female rats by the dose of ryanodine shown to be effective (1 pg; Fig. 3a). In addition, when a higher dose of ryanodine, 100 ng (which was the minimum dose able to induce priming in male rats), was injected in female rats that have been treated with the ODN AS to ERα, we observed prolongation of the PGE_2_-induced hyperalgesia (Fig. 3b), indicating the presence of priming. These results support the suggestion that estrogen, by acting at ERα, sensitizes a ryanodine-dependent mechanism, which might explain the large difference in the dose of ryanodine able to induce priming in male and female rats. Also, the fact that the induction of priming by the higher dose of ryanodine was not affected by the AS ODN treatment suggests that male rats do not express the ERα-dependent mechanism by which the response to ryanodine is amplified, producing priming, and thus needing a higher dose. However, the mechanism downstream from ERα by which estrogen signals in the nociceptor, to regulate the concentration of ryanodine needed to produce hyperalgesic priming, remains to be established. It is also unknown whether this is a direct effect of circulating estrogen on the primary afferent nociceptor ryanodine receptor or an indirect effect of ERα signaling, at another target in the peripheral terminal of the nociceptor.

**Figure 4:**
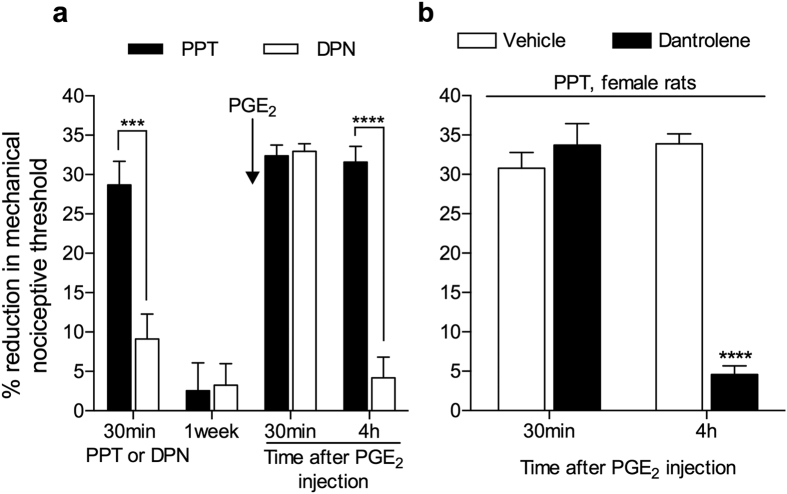
Activation of estrogen receptor alpha (ERα) induces hyperalgesic priming in female rats. (**a**) Female rats received an intradermal injection of PPT, a specific ERα agonist (1 μg, black bars), or DPN, a specific ERβ agonist (1 μg, white bars), on the dorsum of the hind paw. Evaluation of the mechanical thresholds 30 min later showed that PPT, but not DPN, induced significant hyperalgesia (***p < 0.0001). After 1 week, a time point when the mechanical thresholds were not different from before the injection of the estrogen receptors agonists (see Supplementary Table S1), testing for the presence of hyperalgesic priming was performed by injecting PGE_2_ (100 ng) at the same site. We observed significant mechanical hyperalgesia, 30 min after PGE_2_ injection, in both groups. However, when the mechanical nociceptive thresholds were evaluated at the 4^th^ h, only the group previously treated with PPT showed hyperalgesia, indicating that PPT, but not DPN, had induced priming (*F*_1,10_ = 36.33; ****p = 0.0001, when both groups are compared; two-way repeated measures ANOVA followed by Bonferroni *post-hoc* test); (**b**) The ryanodine receptor antagonist dantrolene (1 μg, black bars), or its vehicle (white bars), was injected on the dorsum of the hind paw of female rats. 10 min later, PPT (1 μg) was injected at the same site. 1 week later, testing for the presence of priming was performed by injecting PGE_2_ (100 ng) at the same site as PPT, and evaluating the mechanical nociceptive threshold 30 min and 4 h later (see Supplementary Table S1, for data about the mechanical nociceptive thresholds before injection of PPT and before injection of PGE_2_). In the group treated with vehicle significant mechanical hyperalgesia was observed at both 30 min and 4 h. However, in the group that received dantrolene the hyperalgesia was no longer present at the 4^th^ h, indicating that the priming induced by PPT was dependent on the ryanodine receptor (*F*_1,10_ = 35.94; ****p = 0.0001, when both groups are compared; two-way repeated measures ANOVA followed by Bonferroni *post-hoc* test). (N = 6 paws, all groups).

Since we observed a dependence on ERα in the induction of priming by ryanodine in female rats, we tested if the activation of this receptor would produce priming. The injection of PPT, a selective agonist at ERα, intradermally on the dorsum of the hind paw in females, as opposed to the injection of DPN, an agonist at ERβ, induced mechanical hyperalgesia that was no longer detected one week later, when we tested for the presence of priming, with injection of PGE_2_ at the same site. We observed that the mechanical hyperalgesia induced by PGE_2_ was only prolonged in the paws that had been treated with PPT (Fig. 4a). Next, considering our result indicating that ERα modulates the ryanodine receptor in order to allow its activation to induce priming (Fig. 3a), we evaluated the effect of dantrolene, which is a ryanodine receptor antagonist, on the induction of priming by PPT. We found that the injection of PPT 10 min after the injection of dantrolene at the same site did not produce priming, tested with PGE_2_ 1 week after PPT (Fig. 4b).

Previous results^12^,^20^ and the *in vivo* experiments described above provide evidence for a crucial role of intracellular calcium signaling modulated by a mechanism involving the estrogen receptor (ERα) and the ryanodine receptor. In this context, our current observations show an ERα-dependent marked distinction between the male and female rat, namely an ability of extremely low doses of ryanodine to induce priming in female rats, when compared to the dose in males. To further investigate the mechanisms involved in the interaction between this effect of ryanodine and ERα, we conducted *in vitro* experiments in cultured dorsal root ganglion (DRG) neurons.

Changes in the cytosolic free calcium concentration ([Ca^2+^]_i_) (calcium transients), evaluated by fluorescent calcium imaging and measured as fluorescence ratio (F340/F380), were considered as the response of neurons to drug applications. To evaluate the impact of estrogen in those responses, cultured female rat DRG neurons were incubated for 24–72 h in the presence or absence of β-estradiol (100 nM, used as saturating concentration), and, also, only small DRG neurons (soma diameter <30 μm), which represent predominantly the C-type nociceptive population of primary sensory neurons^32^,^33^ were considered. Additionally, since hyperalgesic priming develops exclusively in IB4+ nociceptors^8^, the cultures were divided into IB4+ and IB4− neurons, to compare the effect of estrogen and ryanodine between these two classes of nociceptors^34^,^35^. In our experiments, the IB4+ and IB4− DRG neurons consisted, respectively, of ~70% and ~30% of the small neurons examined (35 and 16 of 51), in agreement with the proportion reported by other studies^36^.

Initially, a concentration-response (2 fM–20 μM) evaluation of the effect of ryanodine application to the DRG cultures was performed, in order to find the lowest concentration that would produce calcium transients. None of the concentrations tested consistently induced calcium transients, including 0.4 nM, which corresponded to the dose of ryanodine found to induce priming in female rats (1 pg diluted in 5 μl of injection solution, Fig. 1) (also, see Supplementary Fig. S2, for the effect of the concentration 2 nM on DRG neurons).

**Figure 5:**
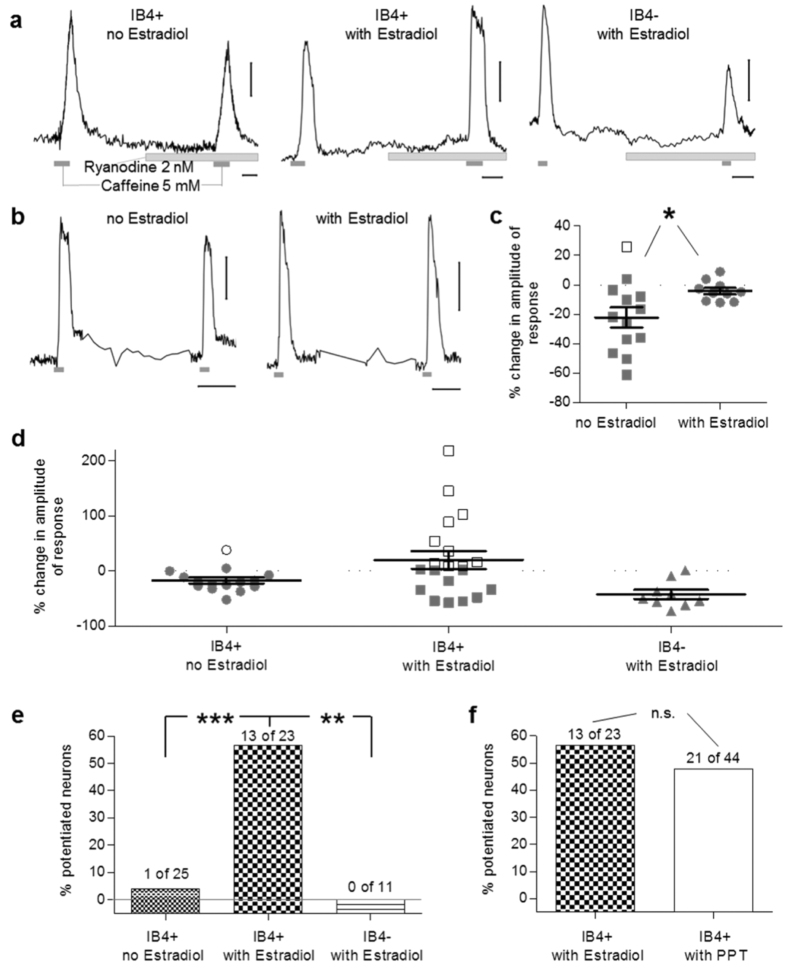
ERα activation is required for potentiation of calcium response in IB4+ small female DRG neurons by low concentration of ryanodine. (**a**) Recordings of [Ca^2+^]_i_ transients in IB4+ and IB4− small DRG neurons, incubated without (left panel) or with (middle and right panels) β-estradiol (100 nM). Ryanodine (2 nM) was applied for 10 min when [Ca^2+^]_i_ returned to baseline after the first application of caffeine (5 mM). After that, caffeine administration was repeated. Whereas no response was produced by ryanodine itself, a significant potentiation of the response to caffeine after ryanodine application occurred, exclusively in IB4+ neurons and predominantly in cultures incubated with β-estradiol; (**b)** Recordings of calcium transients induced by two subsequent applications of caffeine (5 mM) in the absence of ryanodine in IB4+ small DRG neurons incubated without (left panel) or with (right panel) β-estradiol; (**c**) Pooled relative changes in amplitude of the second response to caffeine without ryanodine application, compared as percentage of the first response, in cultures incubated with β-estradiol or vehicle (*p < 0.05, two-tailed unpaired Student’s *t*-test with Welch’s correction: *t*_14_ = 2.56, *p* = 0.02); (**d)** Pooled relative changes in amplitude of the response to caffeine after ryanodine application, compared as percentage of the response before ryanodine application, as described in (**a)**. Potentiated (above the 8% cut-off) and non-potentiated neuron activation are plotted as white and grey symbols, respectively; (**e**) Percentage of potentiated neurons in different groups of cultured neurons (IB4+ without estradiol; IB4+ with estradiol; IB4− with estradiol), compared by Exact Fisher’s test (**p < 0.01, ***p < 0.001, when the IB4+ with estradiol is compared to the other groups: *p* < 0.0001 vs IB4+ without β-estradiol; *p* = 0.0018 vs IB4−). Of note, there is a remarkably higher number of potentiated cells among the IB4+ neurons incubated with β-estradiol; (**f**) Percentage of potentiated IB4+ neurons in culture incubated with β-Estradiol (100 nM) or PPT (100 nM). No significant difference was observed between two groups (*p* = 0.61 > 0.05, exact Fisher’s test). For panels (**a**) and (**b**), the horizontal scale bars correspond to 100 s and, the vertical ones correspond to 0.1 a.u. (arbitrary units of the fluorescence ratio F340/F380).

To establish a sensitizing effect of ryanodine on ryanodine receptors, we evaluated its effect on the calcium transients induced by caffeine, known to directly activate ryanodine receptors^37^. Two subsequent identical short applications of caffeine (5 mM) were used as test stimulus (Fig. 5a,b), prior to (as a control) and after ryanodine application. Changes in the amplitudes of the second responses were used as measure of the sensitizing effect of ryanodine and were compared in IB4+ and IB4− small DRG neurons, incubated in the presence of β-estradiol or its vehicle. We observed that some small DRG neurons showed increased (“potentiated”) response to caffeine, applied after the pretreatment with ryanodine for 10 min, compared to the response in the control neurons (without ryanodine treatment), and the concentration of ryanodine at 2 nM, which is only 5 times higher than the concentration able to induce priming *in vivo* (1 pg in 5 ml, Fig. 1), produced potentiation exclusively in IB4+ neurons, and almost only in the neurons incubated with β-estradiol (Fig. 5a, middle panel). This concentration was chosen in the following experiments. Of note, no significant difference in the amplitudes of the responses to caffeine before ryanodine application was observed among the three groups (first peak, Fig. 5a, *p* = 0.29 > 0.05, one-way ANOVA, *F*_2,60_ = 1.26). Analysis of the changes in amplitude of caffeine-induced responses after ryanodine application showed only 1 out of 13 neurons in the group without β-estradiol potentiated (Fig. 5c, white symbol, ~26% increase in amplitude), which was identified as an outlier in a Gaussian distribution (D’Agostino & Pearson omnibus normality test). Importantly, regarding the effect of β-estradiol in the cultured neurons, it produced significant changes both in terms of means (−22 ± 7%, N = 13, for the group without β-estradiol versus −4 ± 2%, N = 10, for the group with β-estradiol; unpaired Student’s *t*-test with Welch’s correction, *t*_14_ = 2.56, *p* = 0.02 < 0.05) and variances (Fisher’s *F*-test, *F*_12,9_ = 12.45, *p* = 0.0007), i.e., the incubation with β-estradiol significantly decreased the desensitization to the second application of caffeine, but never produced substantial potentiation (Fig. 5c). Based on this analysis, the highest observed value of 8% was established as cut-off for the phenomenon of potentiation, i.e., only changes above this value were evaluated as a potentiated response to caffeine. Thus, regardless of the presence of β-estradiol during culture incubation, no potentiation of the response to caffeine in the absence of ryanodine was observed, suggesting the essential role of a target of ryanodine in the phenomenon.

In contrast, after application of ryanodine (2 nM), a remarkably higher number of cells showed a potentiated (above the 8% cut-off) response to the second caffeine application, specifically among IB4+ neurons incubated with β-estradiol (Fig. 5d). As a side note, similar to what we observed in the data in Fig. 5c, 1 (white symbol) out of 25 neurons in the group without β-estradiol demonstrated spontaneous potentiation (~38% of amplitude) and was also identified as an outlier in the Gaussian distribution. However, in the group of IB4+ neurons incubated with β-estradiol, the distribution of relative changes in amplitude was not Gaussian (*p* = 0.0051 < 0.05, D’Agostino & Pearson omnibus normality test, *K*2 = 10.55) and was significantly different from the distributions in the two other groups (*p* < 0.05, two-sample Kolmogorov-Smirnov test: *p* = 0.012, D = 0.52 vs IB4+ without β-estradiol; *p* = 0.004, D = 0.62 vs IB4−), providing a basis for splitting this group into potentiated (white symbols) and non-potentiated (gray symbols) cells (Fig. 5d, middle group). The values for potentiated and non-potentiated neurons separately passed normality tests (Shapiro-Wilk and D’Agostino & Pearson omnibus normality tests). We found no significant difference between non-potentiated neurons within the three groups (*p* = 0.07 > 0.05, single-way ANOVA, *F*_2,30_ = 2.85). The average magnitude of increase in the potentiated subgroup of IB4+ neurons incubated with β-estradiol was 69 ± 22% (N = 10, Fig. 5d, middle group). Interestingly, 3 of the IB4+ neurons incubated with β-estradiol that responded to caffeine after ryanodine application were insensitive to a previous application of caffeine, showing a transition from insensitive to responsive in these cells. Collectively, we observed potentiation in the response to caffeine after ryanodine application in more than 50% neurons in the “IB4+ with Estradiol” group (Fig. 5e, middle bar). Also, the percentage of potentiated neurons was significantly higher among IB4+ neurons incubated with β-estradiol when compared to those incubated without β-estradiol or to IB4− neurons (*p* < 0.05, exact Fisher’s test: *p* < 0.0001 vs IB4+ without β-estradiol; *p* = 0.0018 vs IB4−). These results suggest that activation of estrogen receptors, specifically in IB4+ neurons, allows the potentiating action of ryanodine.

Finally, we tested if activation of ERα alone could reproduce the effect of β-estradiol. We found that the percentage of potentiated IB4+ neurons incubated with the selective ERα agonist PPT (100 nM, Fig. 5f) as well as the average magnitude of potentiation (41 ± 11%, N = 21) were not significantly different from that observed in the cultures incubated with β-estradiol [*p* > 0.05, exact Fisher’s test (*p* = 0.61) and unpaired Student’s *t*-test (*t*_29_ = 1.25; *p* = 0.22), respectively for the percentage and the magnitude]. Even so, the percentage was still significantly higher than one observed in cultures incubated without PPT or β-estradiol (*p* = 0.0001, Exact Fisher’s test), thus confirming the important role of ERα activation in the potentiation of the response to caffeine in the presence of ryanodine.

## Discussion

We evaluated for sexual dimorphism in the mechanism underlying hyperalgesic priming, downstream of inducing cell surface receptors and proximal second messenger, PKCε^12^,^13^,^20^. Inflammatory mediators and neurotrophic factors, acting at their cognate receptors on the nociceptor, and activation of PKCε, a common downstream second messenger in the peripheral terminal of the nociceptor, induce hyperalgesic priming in male and gonadectomized female, but not in gonad-intact female rats^13^. Importantly, this sexual dimorphism in hyperalgesic priming was not due to lack of cytokine and growth factor action at their cognate receptors on the primary afferent nociceptor, as their intradermal injection, or the intradermal injection of a direct activator of PKCε that does not produce hyperalgesic priming in female rats, does produce a robust acute mechanical hyperalgesia in female as well as male rats^13^. Given the *female* predominance observed in many chronic pain syndromes^1^,^2^,^5^,^38^, we performed the present studies to determine if sexual dimorphism was present when priming was induced by activation of second messengers signaling downstream of PKCε. Since ryanodine, which induces hyperalgesic priming by acting downstream of PKCε^12^,^20^, can stimulate release of calcium from the endoplasmic reticulum^39^, and calcium handling is sexually dimorphic in several cell types^40^, we examined for sexual dimorphism in ryanodine-dependent calcium signaling^40^,^41^. Quite unexpectedly, female rats were markedly more sensitive to ryanodine than males, for the induction of priming. And, while calcium handling in diverse cell types is sexually dimorphic^40^, some in an estrogen/estrogen receptor-dependent manner^42^, and more often greater in females^42^, these sex differences are considerably less dramatic than observed in the sexual dimorphism of the ability of ryanodine to induce hyperalgesic priming (Fig. 1).

To confirm that the hyperalgesic priming induced in male and female rats by ryanodine was mediated by the release of calcium from the stores in the endoplasmic reticulum, we pre-treated rats with an inhibitor of the calcium pump in the intracellular membranes located in endoplasmic reticulum, thapsigargin, at the site of nociceptive testing, as described previously^20^. Thapsigargin, as well as the ryanodine receptor antagonist dantrolene, prevented the induction of priming by ryanodine, supporting the suggestion that the ability of ryanodine to induce priming is dependent on an increase in intracellular calcium, released from the endoplasmic reticulum (Fig. 2). That said, administration of a calcium ionophore, which increases the intracellular calcium concentration^43^, induced mechanical hyperalgesia, but not hyperalgesic priming. Although this effect of the calcium ionophore was dependent on calcium, since it was inhibited by the calcium chelator TMB-8, it did not involve the release of calcium from the stores in the endoplasmic reticulum, as thapsigargin did not prevent it (see Supplementary Fig. S4). Together, these results indicate that an increase in calcium concentration in the cytoplasm might not be enough to produce priming, and support the importance of the endoplasmic reticulum in the activation of the mechanisms that ultimately produce the neuroplastic changes in the nociceptor.

We have previously reported that the sexual dimorphism in hyperalgesic priming is estrogen dependent^13^. Therefore, we next determined if either of the two classic estrogen receptors, ERα and/or ERβ, both of which are present in the primary afferent nociceptor^23^,^24^, specify the observed estrogen-dependence. We found that intrathecal treatment with ODN antisense for ERα mRNA prevented the induction of priming by low-dose, but not by high-dose, ryanodine in female rats (Fig. 3). This result supports the hypothesis that ERα plays a role as modulator of the response to ryanodine, perhaps amplifying it, allowing a very small dose to induce priming. Also in line with this idea, the agonist of ERα, but not of ERβ, induced priming in female rats, in a ryanodine receptor dependent manner (Fig. 4), thus establishing the pathway ERα-ryanodine receptor-calcium release in the development of hyperalgesic priming in females, a mechanism not present in males (see Supplementary Fig. S2), explaining the need of a more intense stimulus (higher dose of ryanodine) to produce neuroplastic change. Of note, ERα is found in the IB4+ population of nociceptors^44^,^45^, which are the neurons susceptible to be primed^8^. In this regard, we have previously demonstrated that the depletion of IB4+ neurons eliminated the induction of hyperalgesic priming^8^, establishing this population of small DRG neurons as responsible for the prolongation of the hyperalgesia induced by PGE_2_ observed in this model of neuroplasticity. However, the mechanism downstream of ERα, by which circulating estrogen acts to regulate the response to ryanodine in IB4+ nociceptors remains to be established.

Our findings from the behavioral experiments were complemented by *in vitro* demonstration that calcium transients produced by caffeine were affected by ryanodine, which sensitizes ryanodine receptors at low concentrations (<10 nM), as previously shown^46^,^47^,^48^, rather than release calcium from the endoplasmic reticulum, in neurons cultured in the presence of β-estradiol. The fact that a higher dose of ryanodine was necessary to induce priming in male rats indicates that the ryanodine-activated mechanism is sensitized/regulated by the higher levels of circulating estrogen in females, as previously suggested^49^, and also indirectly shown *in vitro* by the potentiation, by ryanodine in the presence of β-estradiol or the ERα agonist PPT, of the effect of caffeine in female DRG neurons (Fig. 5). Thus, since the sensitization of ryanodine receptors through the estrogen receptor is not present in males (see Supplementary Fig. S2, showing no potentiation of calcium transients in male DRG neurons, even in presence of β-estradiol), a higher dose of ryanodine is needed to trigger the mechanism downstream from the receptor that ultimately produces priming (Fig. 1). It is also important to emphasize that this potentiation of the responses to caffeine by ryanodine in the presence of estrogen/PPT was observed only in IB4+ small DRG neurons, which is the population of nociceptors that mediate priming^8^, and not observed when ryanodine was not applied to the cultures, ruling out an effect of β-estradiol by itself. Thus, our *in vitro* and *in vivo* observations strongly support the suggestion that what we see in terms of sexual dimorphism in hyperalgesic priming is estrogen dependent, negatively regulating the female nociceptor to be primed by activation of PKCε^13^, but sensitizing the response to ryanodine.

In conclusion, we provide evidence for a profound, ERα-dependent, sexual dimorphism in ryanodine-induced hyperalgesic priming (i.e., females being dramatically more sensitive than males to hyperalgesic priming induced by ryanodine), which is opposite to the direction of the previously demonstrated sexual dimorphism (i.e., male sensitive, female completely insensitive^13^) observed when priming is induced by activation of cell surface receptors or proximal second messengers, such as PKCε, that signal through the same downstream second messengers, the ryanodine receptor, and αCaMKII^12^,^20^. However, additional investigation is needed to determine the mechanisms involved in this dual role of estrogen in females and its possible clinical relevance for the induction of chronic pain.

## Methods

### Animals

All experiments were performed on adult male and female Sprague-Dawley rats (220–400 g; Charles River Laboratories, Hollister, CA). Rats were housed three per cage, under a 12 h light/dark cycle, in a temperature- and humidity-controlled animal care facility at the University of California, San Francisco. Food and water were available *ad libitum*. Nociceptive testing was done between 10:00 am and 5:00 pm. The experimental protocols were approved by the Institutional Animal Care and Use Committee at the University of California at San Francisco, and adhered to the National Institutes of Health Guidelines for the Care and Use of Laboratory Animals. Effort was made to minimize the number of animals used and their suffering.

### Testing mechanical nociceptive threshold

Mechanical nociceptive threshold was quantified using an Ugo Basile Analgesymeter^®^ (Randall-Selitto paw-withdrawal test, Stoelting, Chicago, IL), which applies a linearly increasing mechanical force to the dorsum of the rat’s hind paw, as previously described^50^,^51^. Rats were placed in cylindrical acrylic restrainers designed to provide adequate comfort and ventilation, allow extension of the hind leg from the cylinder, and minimize restraint stress. To acclimatize rats to the testing procedure, they were adapted to the restrainer for 1 h prior to starting each study and for 30 min prior to experimental manipulations. The nociceptive threshold was defined as the force, in grams, at which the rat withdrew its paw. Baseline paw-pressure nociceptive threshold was defined as the mean of the three readings taken before the test agents were injected. Each paw was treated as an independent measure and each experiment performed on a separate group of rats.

### Drugs and reagents

The drugs used in this study were caffeine, a ryanodine receptor agonist; prostaglandin E_2_ (PGE_2_), a direct-acting hyperalgesic agent; ryanodine, a ryanodine receptor modulator; dantrolene sodium salt, a ryanodine receptor inhibitor; 1,3,5-Tris(4-hydroxyphenyl)-4-propyl-1H-pyrazole [PPT, a specific ERα agonist.]; 2,3-Bis(4-hydroxyphenyl)propionitrile [DPN, a specific ERβ agonist]; thapsigargin, an endoplasmic reticulum calcium pump inhibitor^30^,^31^; calcium ionophore A23187, which increases intracellular calcium levels^43^; the inhibitor of calcium influx 3,4,5-trimethoxybenzoic acid 8-(diethylamino) octyl ester (TMB-8)^52^; and, β-estradiol-Water Soluble (cyclodextrin-encapsulated 17β-estradiol), an estrogen receptor (ER) agonist, all from Sigma-Aldrich (St. Louis, MO); Griffonia simplicifolia isolectin B4 (IB4) conjugated to Alexa Fluor^®^ 488 dye (Invitrogen Life Technologies, Carlsbad, CA); and, fura-2 acetoxymethyl ester (fura-2 AM), a membrane permeable form of the fluorescent calcium indicator Fura-2 (Calbiochem, La Jolla, CA). Selection of drug doses was based on our previous studies^12^,^20^,^53^,^54^. The required drug concentrations were achieved by dilutions in 0.9% NaCl (for *in vivo* experiments) or in external perfusion solution (for *in vitro* experiments).

Solutions of β-estradiol, TMB-8 and caffeine, dissolved in 0.9% NaCl, were freshly prepared. Stock solutions of PGE_2_ in absolute ethanol (1 μg/μl) were diluted in 0.9% NaCl (1:50, C_final_ = 0.2 μg/μl) immediately before injection. The ethanol concentration of the final PGE_2_ solution was ∼2% and the injection volume 5 μl. Ryanodine was also first prepared as a stock solution, in absolute ethanol, and then diluted in 0.9% NaCl to the required concentration, depending on the dose needed. Dantrolene and calcium ionophore were dissolved in DMSO and further diluted in 0.9% NaCl containing 10% DMSO; stock solutions of PPT, DPN, thapsigargin and fura-2 AM (1 mM) were prepared in 100% DMSO, and diluted in 0.9% NaCl containing 10% DMSO at the time of the experiments; IB4 was prepared as a stock solution (1 μg/μl), in calcium- and magnesium-free PBS (Invitrogen Life Technologies). Importantly, the concentrations of DMSO or ethanol used to dissolve/dilute the reagents in this study produced no nociceptive effects when tested *in vivo* (see Supplementary Fig. S3, for control experiments).

In the behavioral experiments, drugs were administered intradermally on the dorsum of the hind paw via a beveled 30-gauge hypodermic needle that was attached to a Hamilton^®^ microsyringe (Reno, NV) by polyethylene (PE-10) tubing. The administration of ryanodine, dantrolene, thapsigargin or TMB-8 was preceded by hypotonic shock to facilitate cell permeability to these agents (2 μl of distilled water, separated by an air bubble, to avoid mixing in the same syringe), to get compounds into the nerve terminal^55^,^56^.

### Induction of hyperalgesic priming

The procedure to induce hyperalgesic priming was based on a previously described protocol^12^,^20^. The inducer of priming (ryanodine or PPT, in this study) was injected intradermally on the dorsum of the hind paw, at the site of nociceptive testing. The presence of priming was confirmed, 3–5 days later, depending on the experiment, by the injection of PGE_2_ (100 ng), at the same site. The mechanical hyperalgesia induced by injection of PGE_2_ in the previously untreated naïve control paw lasts no more than 2 h^57^, and in this study is represented by the reduction in the mechanical threshold evaluated 30 min after PGE_2_ injection, which expresses its acute hyperalgesic effect, even in the previously primed paw^11^,^12^; The prolongation of PGE_2_ hyperalgesia to greater than 4 h is used as a marker for the presence of priming^7^,^10^,^12^,^58^, produced by activation of an additional signaling pathway^9^,^11^,^12^, and represented as the reduced mechanical threshold at the 4^th^ h time point^12^. Importantly, immediately before the tests for priming with PGE_2_, the mechanical nociceptive threshold is not significantly different from the mechanical baseline threshold evaluated before the injection of the priming inducer (3–5 days previously, see Supplementary Table S1).

### ODN AS to ERα and ERβ mRNA

To investigate the role of estrogen receptor subtypes in hyperalgesic priming induced by ryanodine, antisense AS ODN against ERα and ERβ mRNA were administered to female rats. The sequence for the ERα, 5′-CAT-GGT-CAT-GGT-CAG-3, and the ERβ, 5′-GAA-TGT-CAT-AGC-TGA-3′, AS ODN (Invitrogen Life Technologies), were directed against unique regions of each rat ER subtype [GeneBank accession numbers NM_012689.1 (ERα) and NM_012754.1 (ERβ)], and have been previously shown to attenuate cellular levels of the respective ERs^59^,^60^. The MM ODN sequences, 5′-ATC-GTG-GAT-CGT-GAC-3′, for ERα, and 5′-AAG-GTT-ATC-GCA-AGT-3′, for ERβ, were scrambled AS ODN sequences that have the same base pairs and GC ratio, with the order randomized, and little or no homology to any mRNA sequences posted at GenBank.

Before use, ODNs were reconstituted in nuclease-free 0.9% NaCl, and then administered intrathecally at a dose of 2 μg/μl in a volume of 20 μl, for 3 consecutive days, starting 3 days before the injection of ryanodine, and then continued for 3 additional days, at which time the evaluation for the presence of priming was performed by intradermal administration of PGE_2_ on the dorsum of the hind paw. As described previously^53^, rats were anesthetized with isoflurane (2.5% in O_2_), and the ODN injected using a microsyringe (10 μl) with a 30-gauge needle, inserted into the subarachnoid space, between the L_4_ and L_5_ vertebrae.

### Preparation of cultures of DRG neurons

Primary cultures of rat sensory neurons were obtained from adult female DRG and prepared as described previously^54^. In brief, under isoflurane anesthesia, rats were decapitated, the dorsum of the vertebral column was then opened, and the L_4_ and L_5_ DRGs rapidly removed, chilled in Hanks’ balanced salt solution (HBSS) on ice and desheathed. Ganglia were treated with 0.125% collagenase P (Worthington Biochemical Corporation, Lakewood, NJ) in HBSS for 90 min at 37 °C, and then treated with 0.25% trypsin (Worthington Biochemical Corporation) in calcium- and magnesium-free PBS (Invitrogen Life Technologies) for 10 min, followed by 3 times washout and trituration in Neurobasal-A medium (Invitrogen Life Technologies) to produce a single-cell suspension. The suspension was centrifuged at 1000 RPM for 3 min and re-suspended in Neurobasal-A medium supplemented with 50 ng/ml nerve growth factor, 100 U/ml penicillin/streptomycin and B-27 (Invitrogen Life Technologies). In some experimental series, the medium was additionally supplemented with β-estradiol (100 nM) or the ERα agonist PPT (100 nM) for activation of estrogen receptors. Cells were then plated on cover slips and incubated at 37 °C in 5% CO_2_ for at least 24 h before use.

### *In vitro* recordings

Cultured DRG neurons were used for *in vitro* experiments between 24 and 96 h after dissociation and plating. Small, medium and large sized neurons were routinely observed in the same preparation, but this study was focused only on cells with a soma diameter less than 30 μm (small DRG neurons, predominantly representing the C-type nociceptor subpopulation). After mounting to a recording chamber the culture medium was replaced with Tyrode’s solution containing 140 mM NaCl, 4 mM KCl, 2 mM MgCl_2_, 2 mM CaCl_2_, 10 mM glucose, 10 mM HEPES, and adjusted to pH 7.4 with NaOH. Tyrode’s solution was used in the further *in vitro* experiments as external perfusion solution and all fluorescent dyes, stimulating and modulating drugs were applied diluted in this solution. The volume of the recording chamber was 150 μl. The perfusion system was gravity-driven at a flow rate of 1–2 ml/min. All experiments were performed at room temperature of 20–23 °C.

### Calcium imaging

The bright-field imaging system consisted of an inverted microscope (Eclipse TE-200, Nikon) with epi-fluorescence attachment, using a mercury lamp for excitation. Illumination was controlled by a Lambda 10-2 filter wheel controller and Lambda SC Smart Shutter controller (Sutter Instruments Co., Novato, CA); an Andor Clara Interline CCD camera (Andor Technology Ltd., Belfast, UK) was used for high-resolution digital image acquisition. MetaFluor software (Molecular Devices LLC, Sunnyvale, CA) provided computer interface and controlled the whole system as well as being used for image processing. A Plan Fluor objective (20xUV, NA 0.50; Nikon) was used for both fluorescent and transmitted light imaging with phase contrast. Calcium imaging was performed using the fluorescent calcium indicator fura-2 acetoxymethyl ester (fura-2 AM) as previously described^54^. Briefly, neurons were loaded with 5 μM fura-2 AM by incubation for 20 min directly in the recording chamber. Then cells were perfused with Tyrode’s solution for 10 min before the beginning of the recording to allow for complete de-esterification of the fura-2 AM. Measurement of the concentration of free calcium ions ([Ca^2+^]_i_) was performed by ratiometric imaging. Fluorescence was excited at 340 and 380 nm for 2–10 ms each, and the emitted light was long filtered at 520 nm using a standard Fura-2 filter set (Chroma Technology, Bellows Falls, VT). Using MetaFluor software (Molecular Devices LLC, Sunnyvale, CA) corresponding pairs of digital images were acquired every 1–10 s (depending on the rate of the examined process, to minimize UV exposure and excitotoxicity); the fluorescence ratio (F340/F380) was calculated on a pixel-by-pixel basis with background correction and averaged for the region of interest defined for each neuron. The fluorescence ratio was used to characterize [Ca^2+^]_i_ without recalculation into concentration. The amplitude of response was measured as the difference between fluorescence ratios at the peak and the base of the responses.

### Histochemistry

Cells were incubated in the dark in Tyrode’s solution supplemented with 10 μg/ml IB4 conjugated to Alexa Fluor^®^ 488 dye (Invitrogen Life Technologies) for 10–12 min. After washout fluorescent images were captured during the first 15 min of each experiment (before prolonged calcium imaging) using a standard GFP filter set (Chroma Technology, Bellows Falls, VT). Cells demonstrating bright fluorescence and halo around the neuronal plasma were considered as IB4−positive (IB4+), whereas those having intensity below 20% of maximum for selected field of view were considered as IB4−negative (IB4−).

### Data analysis

All behavioral data are presented as mean ± standard error of the mean (SEM) of N independent observations. Statistical comparisons were made using GraphPad Prism 5.0 statistical software (GraphPad Software, Inc., La Jolla, CA). A *p*-value < 0.05 was considered statistically significant. In behavioral experiments, the dependent variable was change in mechanical paw-withdrawal threshold, expressed as the percentage change from baseline. No significant difference in the mechanical nociceptive thresholds was observed before the injection of the priming stimuli (ryanodine or PPT) and immediately before injection of PGE_2_ (average mechanical nociceptive threshold before priming stimuli: 121.7 ± 0.8 g; average mechanical nociceptive threshold before PGE_2_ injection: 121.1 ± 0.7 g; N = 168 paws; paired Student’s *t*-test, *t*_167_ = 1.014, *p* = 0.3119). As specified in the figure legends, Student’s *t*-test or two-way repeated-measures analysis of variance (ANOVA), followed by Bonferroni *post-hoc* test, was performed to compare the magnitude of the hyperalgesia induced by the priming stimulus or by PGE_2_ injection with the control groups, or to compare the effect produced by the different treatments on the prolongation of the PGE_2_-induced hyperalgesia, with the control groups, at the determined time points. Of note, in Fig. 1, the mechanical thresholds at the 4^th^ h after PGE_2_ injection were compared with the respective baseline thresholds before the injection of PGE_2_, in order to evaluate if the mechanical hyperalgesia was present at that time point.

Calcium imaging results are presented as changes in amplitude of the responses to drug application, calculated for each cell as the percentage of the amplitude of its initial (premodulated) response. Shapiro-Wilk’s and D’Agostino and Pearson’s omnibus normality tests were used to determine if distributions were Gaussian. If not, the two-sample Kolmogorov-Smirnov test was applied to elucidate differences between distributions. Differences between two normally distributed groups were analyzed using two-tailed unpaired Student’s *t*-test for unequal variances (Welch correction) for means, and Fisher’s test for variances, whereas one-way ANOVA was used in case of multiple treatments. Exact Fisher’s test for contingency tables was performed to analyze changes in the proportions of DRG neurons within a category (responsive, “potentiated”, etc.).

## Additional Information

**How to cite this article**: Ferrari, L. F. *et al*. Marked Sexual Dimorphism in the Role of the Ryanodine Receptor in a Model of Pain Chronification in the Rat. *Sci. Rep.* **6**, 31221; doi: 10.1038/srep31221 (2016).

## Supplementary Material

Supplementary Information
