# Supplementary material for: Marked Sexual Dimorphism in the Role of the Ryanodine Receptor in a Model of Pain Chronification in the Rat
Source: Sci Rep. 2016 Aug 8;6:31221. doi: 10.1038/srep31221 (PMC4976309; doi:10.1038/srep31221)
Supplement: Supplementary Information [file srep31221-s1.pdf]

Marked Sexual Dimorphism in the Role of the Ryanodine Receptor in a Model of Pain Chronification in the Rat

Luiz F. Ferrari[1], Eugen V. Khomula[1], Dionéia Araldi[1], and Jon D. Levine[1]*

[1] Departments of Medicine and Oral Surgery, and Division of Neuroscience, University of California at San Francisco, 521 Parnassus Avenue, San Francisco, CA 94143, USA

Supplementary Information

Supplementary Table S1: Mechanical nociceptive thresholds before the injection of the priming inducer and before the injection of PGE_2

The mechanical nociceptive thresholds, evaluated by the Randall-Selitto paw withdrawal test, before the injection of the priming stimulus (ryanodine or PPT) and before the testing for presence of priming by injecting PGE_2 (100 ng) at the same site, for each experimental group, are shown. Comparison of these two values showed no significant difference (NS, paired Student's *t*-test) in all groups. (N = 6 paws per group)

FIGURE	GROUP	Average mechanical nociceptive threshold before the injection of the priming inducer	Average mechanical nociceptive threshold before the injection of PGE_2	Comparison (paired Student's *t*-test, NS for all groups)
**Fig. 1**	Ryanodine, 0.1 pg, female	122.6 ± 3.9 g	121.0 ± 3.6 g	t_5 = 1.536; p = 0.1852
	Ryanodine, 1 pg, female	116.3 ± 3.7 g	118.0 ± 3.3 g	t_5 = 0.5951; p = 0.5777
	Ryanodine, 10 pg, female	121.0 ± 2.2 g	119.6 ± 4.0 g	t_5 = 0.5680; p = 0.5946
	Ryanodine, 30 pg, female	116.6 ± 1.7 g	116.0 ± 2.0 g	t_5 = 0.2162; p = 0.8374
	Ryanodine, 100 pg, female	117.6 ± 1.7 g	120.0 ± 3.6 g	t_5 = 0.7488; p = 0.4877
	Ryanodine, 10 ng, female	119.3 ± 3.1 g	120.3 ± 2.4 g	t_5 = 0.3384; p = 0.7488
	Ryanodine, 100 pg, male	121.3 ± 2.9 g	119.3 ± 1.7 g	t_5 = 0.7906; p = 0.4650
	Ryanodine, 10 ng, male	118.3 ± 2.0 g	119.6 ± 2.0 g	t_5 = 0.5976; p = 0.5761
	Ryanodine, 30 ng, male	118.0 ± 4.1 g	117.0 ± 2.1 g	t_5 = 0.2335; p = 0.8246
	Ryanodine, 100 ng, male	121.3 ± 0.8 g	119.6 ± 2.8 g	t_5 = 0.5141; p = 0.6291
**Fig. 2**	panel a, males, control	121.3 ± 0.8 g	119.6 ± 2.8 g	t_5 = 0.5141; p = 0.6291
	panel a, males, dantrolene	120.0 ± 2.5 g	122.0 ± 1.0 g	t_5 = 4.140; p = 0.9090
	panel a, females, control	116.3 ± 3.7 g	118.0 ± 3.3 g	t_5 = 0.595; p = 0.5777
	panel a, females, dantrolene	124.0 ± 2.0 g	123.6 ± 2.4 g	t_5 = 0.2548; p = 0.8090
	panel b, males, control	121.3 ± 0.8 g	119.6 ± 2.8 g	t_5 = 0.5141; p = 0.6291
	panel b, males, thapsigargin	128.3 ± 4.0 g	127.6 ± 2.4 g	t_5 = 0.1932; p = 0.8544
	panel b, females, control	116.3 ± 3.7 g	118.0 ± 3.3 g	t_5 = 0.5951; p = 0.5777
	panel b, females, thapsigargin	130.0 ± 2.5 g	120.6 ± 1.3 g	t_5 = 4.427; p = 0.2068
**Fig. 3**	Panel a, ERα mismatch group	126.6 ± 2.1 g	130.0 ± 0.8 g,	t_5 = 2.076; p = 0.0925
	Panel a, ERα antisense group	127.8 ± 2.2 g	132.3 ± 3.5 g	t_5 = 1.958; p = 0.1076
	Panel a, ERβ mismatch group	123.3 ± 3.3 g	124.3 ± 2.4 g	t_5 = 0.3744; p = 0.7234
	Panel a, ERβ antisense group:	120.0 ± 2.5 g	20.3 ± 3.9 g	t_5 = 0.1190; p = 0.9099
	Panel b, ERα mismatch group	125.0 ± 3.4 g	121.0 ± 2.1 g	t_5 = 1.257; p = 0.2644
	Panel b, ERα antisense group	120.0 ± 3.6 g	120.6 ± 2.2 g	t_5 = 0.1832; p = 0.8618
**Fig. 4**	Panel a, PPT group	123.6 ± 3.2 g	120.0 ± 2.0 g	t_5 = 0.8584; p = 0.4299
	Panel a, DPN group	131.3 ± 2.1 g	127.0 ± 3.8 g	t_5 = 1.182; p = 0.2904
	Panel b, vehicle-treated group	112.6 ± 3.1 g	117.0 ± 2.1 g	t_5 = 1.337; p = 0.2389
	Panel b, dantrolene-treated group	118.6 ± 1.6 g	119.6 ± 2.8 g	t_5 = 0.4060; p = 0.7015

Supplementary Figure S1: Injection of ryanodine does not induce mechanical hyperalgesia

Different groups of female and male rats received intradermal injection of different doses of ryanodine (females: 0.1 pg; 1 pg; 10 pg; 30 pg; 100 pg; 10 ng; males: 100 pg; 10 ng; 30 ng; 100 ng) on the dorsum of the hind paw. Mechanical nociceptive threshold was evaluated before and 30 min and 5 days after the ryanodine injection, immediately before the tests for the presence of hyperalgesic priming by injecting PGE_2 at the same site. Two-way repeated measures ANOVA followed by Bonferroni *post-hoc* test showed no significant difference in the mechanical nociceptive threshold among the different groups, even considering the three time points analyzed ($F_{2,10}$ = 0.02541; p = 0.9750); no difference within each group for the three time points (i.e., the injection of the different doses of ryanodine did not cause changes in the mechanical threshold over time, $F_{9,45}$ = 0.5974; p = 0.7922), and no effect of the treatment (different doses of ryanodine) on the mechanical nociceptive threshold for the three time points ($F_{18,90}$ = 0.3222; p = 0.9957). (N = 6 paws per group)

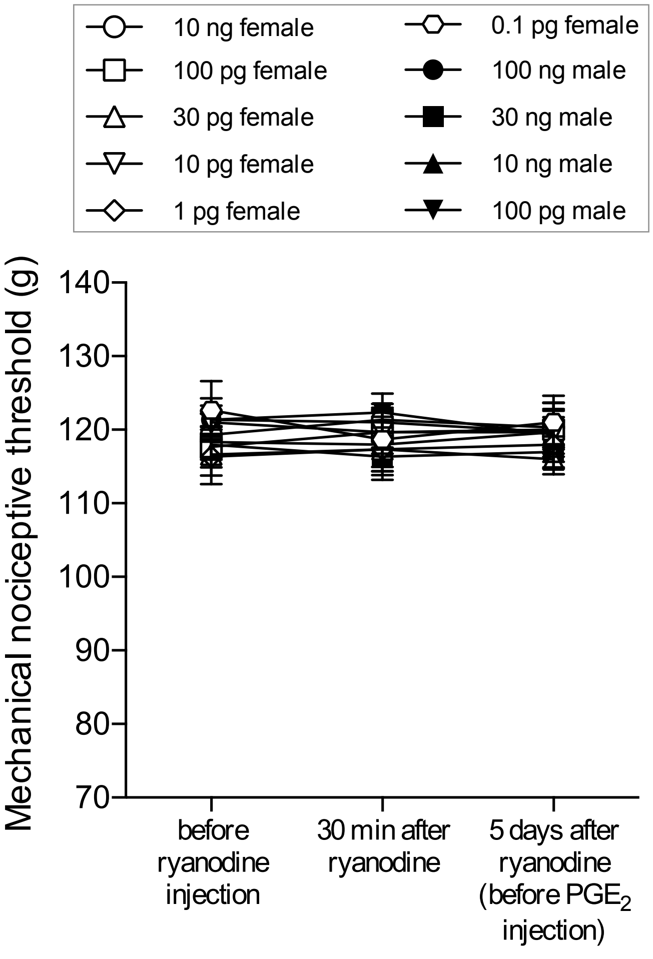

Supplementary Figure S2: Application of ryanodine to male DRG IB4+ neurons in the presence of estradiol does not potentiate calcium transients

Representative recording of $[Ca^{2+}]_i$ transients in an IB4+ DRG neuron from a male rat, incubated with β-estradiol (100 nM). No response was produced by application of ryanodine (2 nM) itself (gray bar), and no potentiation of the response to caffeine was observed after ryanodine application. Paired comparison shows that the means of absolute and relative differences between the amplitudes of the second and the first responses were -0.04 ± 0.02 a.u. and -13 ± 9% (N = 6), respectively. No significant difference versus time zero was found (one-sample two-tailed t-test: $t_5 = 1.51$, $p = 0.19$ and $t_5 = 1.64$, $p = 0.16$, respectively).

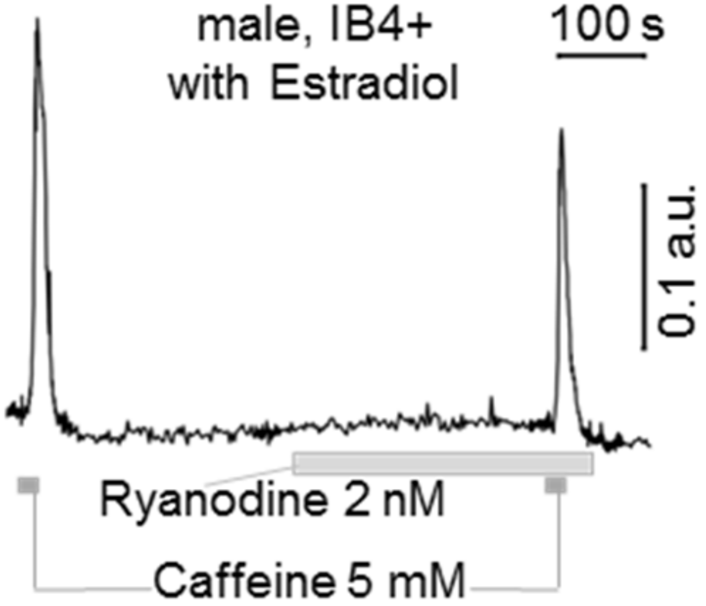

Supplementary Figure S3: Effect of ethanol or DMSO on the mechanical nociceptive threshold

Different groups of rats received an intradermal injection of ethanol (2%, open circles) or DMSO (10%, dark circles), which are vehicles used to dissolve the compounds in this study, on the dorsum of the hind paw. Mechanical nociceptive thresholds were evaluated before and 30 min after the injections. No significant changes were observed after the injection of ethanol or DMSO (ethanol group, $t_5 = 0.4385$, $p = 0.6793$; DMSO group: $t_5 = 1.065$, $p = 0.3355$; when the mechanical nociceptive thresholds before and after the injections are compared, paired Student's *t*-test), showing that the vehicles do not induce mechanical hyperalgesia by themselves. (N = 6 paws per group)

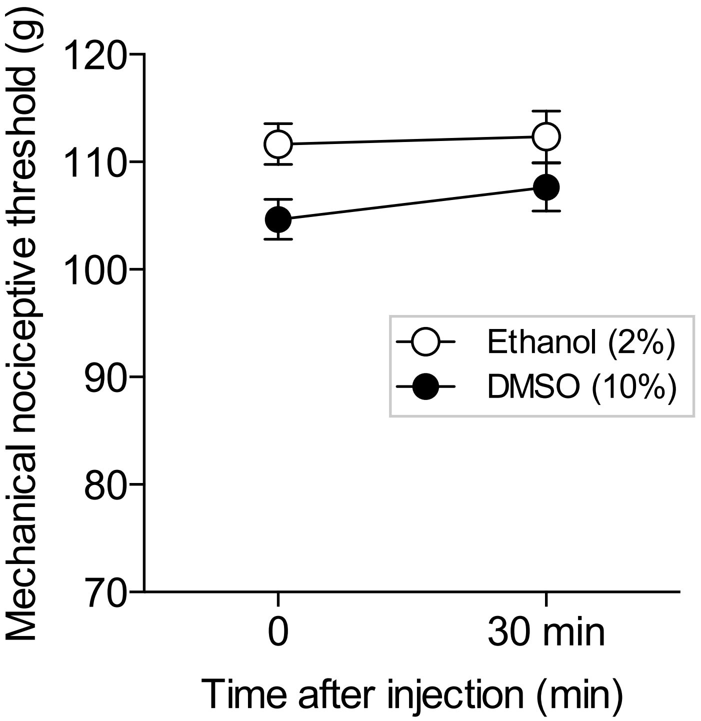

Supplementary Figure S4: Increase of calcium in the cytosol is not enough to induce hyperalgesic priming

a: Calcium ionophore (Ca^{2+} ionoph, 1 μg) was injected on the dorsum of the hind paw of male (left panel) or female (right panel) rats. The mechanical nociceptive threshold was evaluated 30 min later by the Randall-Selitto paw-withdrawal test. Mechanical hyperalgesia was observed in both groups (male group: $t_5 = 8.216$, $p = 0.004$; female group: $t_5 = 12.00$, $p < 0.0001$, when the mechanical nociceptive thresholds before and 30 min after the injections are compared, paired Student's *t*-test). One week later, the presence of priming was evaluated by injecting PGE_2 at the same site where Ca^{2+} ionoph had been injected. Significant mechanical hyperalgesia was observed 30 min after PGE_2 injection. However, at the 4th h the hyperalgesia was no longer present (paired Student's *t*-test comparing the mechanical nociceptive thresholds before and 4 h after PGE_2 injection: male group: $t_5 = 1.655$, $p = 0.1589$, NS; female group: $t_5 = 2.101$, $p = 0.0896$, NS), indicating that Ca^{2+} ionoph did not induce priming; **b:** To evaluate if the mechanical hyperalgesia induced by injection of Ca^{2+} ionoph was due to the release of calcium from the endoplasmic reticulum, in addition to the influx of calcium from the extracellular space, Ca^{2+} ionoph was injected on the dorsum of the hind paws of male (no-pattern bars) or female (dotted bars) rats in the absence (control, white bars) or presence of the calcium chelator TMB-8 (TMB, 1 μg, light gray bars) or the endoplasmic reticulum calcium pump inhibitor thapsigargin (Thaps, 1 μg, darker gray bars). We observed that the magnitude of the mechanical hyperalgesia induced by Ca^{2+} ionoph, evaluated 30 min after injection, was significantly attenuated in the groups pretreated with TMB-8, but not in the groups pretreated with thapsigargin (male groups: $F_{2,15} = 18.12$, *** $p < 0.0001$; female groups: $F_{2,15} = 39.65$, *** $p < 0.0001$, one-way ANOVA followed by Bonferroni *post-hoc* test), indicating that the hyperalgesic effect of Ca^{2+} ionoph does not involve release of calcium channels from the stores in the endoplasmic reticulum. (N = 6 paws per group)

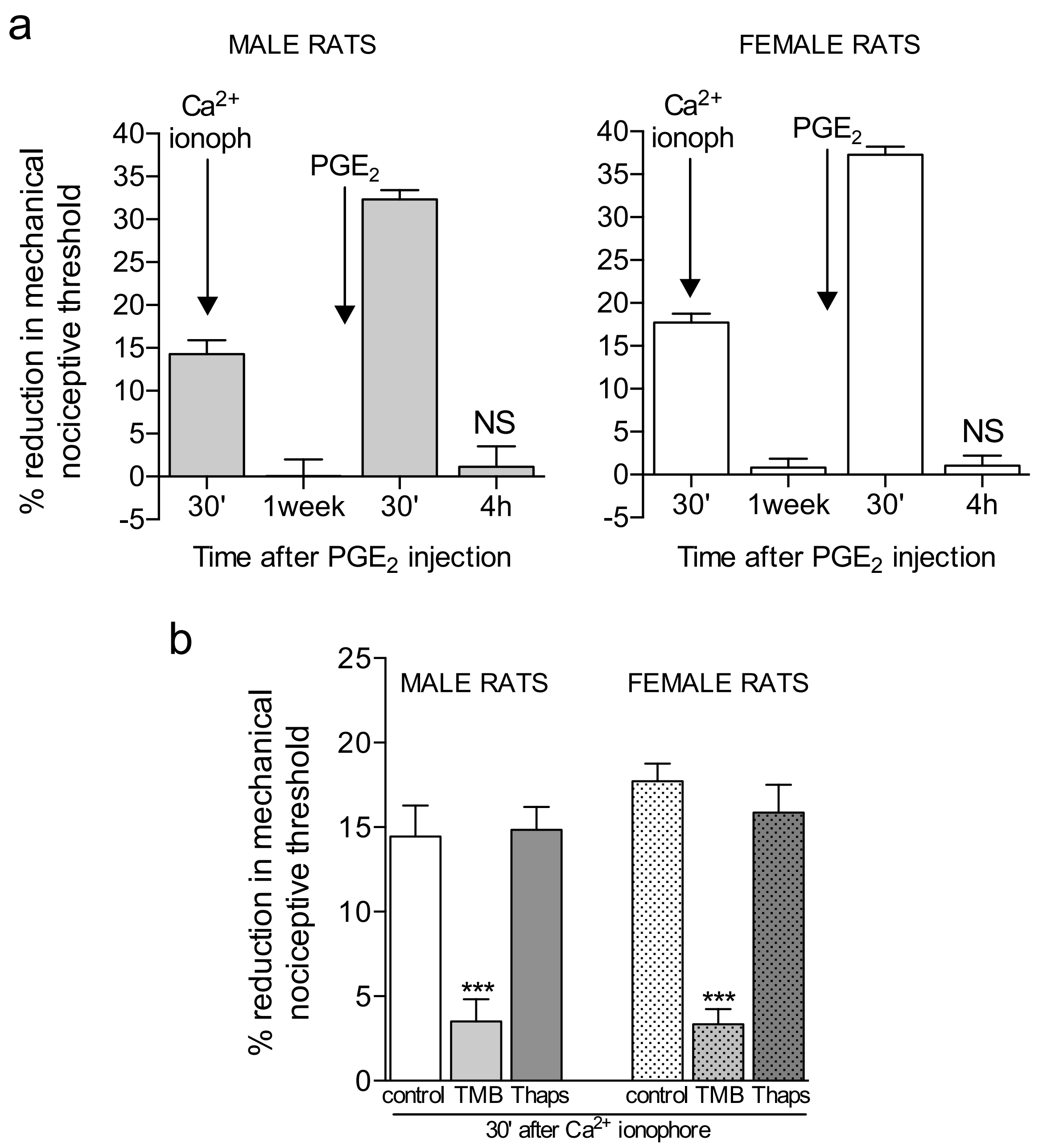
a
MALE RATS
FEMALE RATS
Ca2+ ionoph
PGE2
% reduction in mechanical nociceptive threshold
40
35
30
25
20
15
10
5
0
-5
NS
30'
1week
30'
4h
Time after PGE2 injection
b
MALE RATS
FEMALE RATS
25
20
15
10
5
0
***
control
TMB
Thaps
30' after Ca2+ ionophore
